# Awareness, agreement, adoption and adherence to type 2 diabetes mellitus guidelines: a survey of Indonesian primary care physicians

**DOI:** 10.1186/1471-2296-15-72

**Published:** 2014-04-23

**Authors:** Indah S Widyahening, Yolanda van der Graaf, Pradana Soewondo, Paul Glasziou, Geert JMG van der Heijden

**Affiliations:** 1 Community Medicine Department, Faculty of Medicine Universitas Indonesia, Jl. Pegangsaan Timur 16, Jakarta 10430, Indonesia; 2 Julius Center for Health Sciences and Primary Care, University Medical Center Utrecht, Heidelberglaan 100, 3584 CX Utrecht, The Netherlands; 3 Internal Medicine Department, Faculty of Medicine Universitas Indonesia – Cipto Mangunkusumo Hospital, Jl. Diponegoro 71, Jakarta 10430, Indonesia; 4 Centre for Research in Evidence-Based Practice (CREBP) Faculty of Health Sciences, Bond University, Gold Coast, Queensland 4229, Australia; 5 Department Social Dentistry, Academic Center for Dentistry Amsterdam (ACTA), Gustav Mahlerlaan 3004, 1081 LA Amsterdam, The Netherlands

**Keywords:** Awareness, Adoption, Adherence, Diabetes, Guidelines, General practitioners

## Abstract

**Background:**

To assess the degree of awareness, agreement, adoption and adherence of physicians in Indonesia to type 2 diabetes mellitus guidelines, and their association with characteristics of the responders.

**Methods:**

Questionnaire survey among General Practitioners (GPs) attending the Indonesian Association of Family Practitioners annual conference in November 2012. The proportion of GPs who were aware of, agreed with, adopted and adhered to the seven recommendations in the guidelines (screening for diabetes, diagnosis, lifestyle modification, use of sulfonylurea, target blood glucose, target blood pressure and use of statin) were calculated in the total number of responders.

**Results:**

Of the 399 GPs participating, 383 (89%) were aware of the existence of Indonesian type 2 diabetes guidelines. Awareness for each recommendation varied from 66 to 91%. The recommendation to use a random blood glucose test for diagnosing patients with classic diabetes symptoms had the least awareness (265/399, 66%) and least agreement (163/399, 41%). The recommendation on statin use was the least adopted (192/399, 48%), while the least adherence (7/399, 2%) was found for the recommendation on screening for diabetes for patients with risk factors. Years of practice experience and proportion of diabetes patients seen in their practice were independently related with adherence to statin prescription.

**Conclusions:**

High awareness of the Indonesian type 2 diabetes guideline does not necessary lead to adoption or adherence to recommendations important for outcomes and quality of care. The awareness-to-adherence model helps in identifying barriers for the use of guidelines.

## Background

A study by the World Health Organization (WHO) estimated that the total number of people with diabetes will increase from 171 million in 2000 to 366 million in 2030; mostly in developing countries [1]. This is due to population growth, aging, urbanization, and increasing prevalence of obesity and physical inactivity. The Basic Health Research Survey conducted by the Indonesian Ministry of Health in 2007 involved 24,417 participants living in urban area from all over Indonesia found that the prevalence of diabetes in Indonesia was about 6%, and about two thirds of that percentage are unaware that they have diabetes [2]. Therefore, Indonesia became the seventh largest country with diabetes people in the world [3].

Diabetes mellitus is a complex chronic disease that requires lifelong self-management and continuous medical care to prevent its acute complications and reduce its associated chronic health risks [4]. Type 2 diabetes, which is resulted from a progressive insulin secretory defect on the background of insulin resistance has been recognized as an emerging health problem in Asia Pacific, including Indonesia. On the other hand type 1 diabetes, which is resulted from β-cell destruction is less common in the region [2,4,5]. However, the two decade existence of the Indonesian guidelines on diabetes management seems insufficient to achieve the targets in diabetes control. Currently, about 68% of type 2 diabetes patients being cared in secondary and tertiary hospitals in Indonesia have poor blood glucose control (HbA1c > 7% or >53 mmol/mol) [6].

Guidelines may assist patients and health professionals in achieving optimal management of diabetes. The Indonesian Society of Endocrinology (Perkeni) introduced a guideline on the management and prevention of type 2 diabetes mellitus in 1993, and revised it on regular basis since then [7]. This guideline provides selected recommendations that have been derived from a selection of internationally established guidelines [4,8-10] and consensus of Indonesian experts in endocrinology.

Several surveys have shown that the adherence varies per guideline recommendation [11-15]. Barriers to guideline adherence have been identified, including the inability to access guidelines and physicians’ attitude and belief toward the guidelines [16]. Pathman et al. reported that for the consistency between patient care and guidelines recommendations, physicians must be aware of, agree with, decide to adopt (i.e. decide it is appropriate and feasible to use in their own practice), and adhere to the recommendations (i.e. actually follow them for appropriate patients at the appropriate time) [17,18]. Several studies have been conducted based on this ‘awareness to adherence’ model, yet only one came from developing countries [17].

We would like to know whether this model applies also for a developing country like Indonesia. In this study we explore the degree of general practitioners’ awareness of agreement with, adoption of and adherence to the type 2 diabetes mellitus guidelines in Indonesia, and identify associated physicians’ characteristics.

## Methods

### Questionnaire design and data collection

Based on the evaluation of hypertension guidelines questionnaire by Heneghan et al. [19], we developed a similar questionnaire centered on items in the Consensus on the Management of Type 2 Diabetes Mellitus 2011 of the Indonesian Society of Endocrinology [7]. We included questions on the respondent characteristics: gender, age, specialization, practice duration, type of practice, location of practice, previous participation on type 2 diabetes management training and number and proportion of diabetes patients seen in their practice. According to the guideline recommendations we grouped the questionnaire content into screening, diagnosis, treatment, life-style modification, management of co-morbidities and diabetes complications (Table 1). For the nominal and ordinal response options we followed the Pathman ‘awareness-to-adherence’ model, notably awareness (Yes/no), agreement (Yes/Unsure/No), and adoption (i.e. the recommendation is being followed in general in the appropriate patients; always/more than half/less than half/never). Adherence was assessed with an open ended question about the system responders had in place to promote or monitor the guideline application.

**Table 1:** Recommendations of the Indonesian type 2 Diabetes Mellitus guideline assessed in the questionnaire

	**Statements in the guideline**
Recommendation 1	Screening for type 2 diabetes should be performed in all patients with any of the risk factor listed in the guidelines.
Recommendation 2	In patients with classic DM symptoms, one random blood (plasma) glucose test with result >200 mg/dL is enough to confirm the diagnosis.
Recommendation 3	For newly diagnosed patients, management should be started with meal planning and exercise for 2–4 weeks.
Recommendation 4	Sulfonylurea is the drug of choice for normal and underweight patients.
Recommendation 5	Most patients should achieve Fasting Blood Glucose (FBG) of <100 mg/dL and 2-hour post-prandial Blood Glucose (2-h pp BG) of <140 mg/dL.
Recommendation 6	Blood pressure should be reduced to below 130/80 mmHg.
Recommendation 7	Statin should be prescribed to people with type 2 diabetes who are over 40 years old or have CVD risk.

The questionnaire was pilot tested to five GPs from the Community Medicine Department of the Faculty of Medicine Universitas Indonesia to determine whether the questions were clear, understandable, and in a logical order (face validity). Moreover the same GPs and three endocrinologists who are familiar with the diabetes guideline were asked to criticize the content of the questionnaire (content validity). Based on the results of this pilot, minor changes were made. Further psychometric evaluation of the reliability was not performed.

The final questionnaire was distributed to all physicians attending the Indonesian Association of Family Practitioners annual conference on November 2012 in Jakarta, Indonesia. The questionnaire was put in the delegates pack together with an information leaflet on consent for survey participation. Returning of the self-completed questionnaire by responders was seen as their token of consent. Before handing out their certificate of attendance conference participants were informed about the possibility of participation. The Health Research Ethics Committee of the Faculty of Medicine Universitas Indonesia reviewed and approved the study.

### Analyses and coding of data

We explored the association between respondent characteristics and adherence to each of the seven guideline recommendations. For this we used multivariate logistic regression analysis. To prevent for a type 2 error, only GP characteristics with a univariate p-value of 0.20 or less were selected for such multivariate analyses. The outcome for these data analyses was the number of responders’ adherent to a recommendation. To prevent for spurious findings at least 10 participants adherent to the recommendation were needed for each respondent characteristic included in the multivariable analysis [20]. We used SPSS, version 20.0 for Windows (SPSS Inc., Chicago, IL, USA) for all data analysis.

Responders were classified as ‘unaware of a recommendation’ if they answered “no” on the question on familiarity with that recommendation. Agreement was classified according to whether they agreed or not with the guideline. They were considered to have adopted a guideline when they reported implementing it ‘more than half of the time’. They were considered to adhere to the recommendation when they ‘always’ or ‘more than half of the time’ applied it in clinical practice and specified the system they used to promote or monitor application. The proportions of doctors who were aware of, agreed with, adopted and adhered to each recommendation were calculated over the total number of responders.

Years of practice experience was grouped in 15 years or less and more than 15 years. The Perkeni guideline was firstly introduced in 1993. Therefore we assume that GPs who practiced less than 15 years should be aware of the guideline during their medical training.

We assumed the missing outcome data to represent no awareness, no agreement, no adoption and no adherence. For respondent characteristics with up to 15% missing data, we used conditional imputation, imputing the mean or median. We used mean and median values for imputation since we thought we had not the proper participant characteristic’s to do regression analyses for imputation.

## Results

From the 662 conference participants, 414 questionnaires (63%) were collected. We included 399 (96%) questionnaires from GPs and excluded 3 questionnaires from specialists and 12 from other health care professionals (e.g. nurses and dietician/nutritionists). The missing data for awareness, agreement, adoption and adherence ranged from none to 10%. Adherence to screening had no missing data, while the proportion of missing data for adherence to statin was highest (10%). Three participant characteristics had 5% or more missing data, with a maximum of 15% for number of diabetes patients seen in a week.

Characteristics of the GP responders are presented in Table 2. The higher proportion of them were female (68%), doing a solo (individual) practice (54%), practiced in Java-the most populated island in Indonesia (72%) and had participated in diabetes management training (64%). Three-hundred forty of 383 GPs (89%) were aware of the consensus on the management of type 2 diabetes by the Indonesian Society of Endocrinology.

**Table 2:** Characteristics of the GP responders

**Characteristics**	**n (%)**	**Mean (SD)**	**Min-max**
Years of practice^a^		15.7 (8.8)	0-45 years
Gender^a^			
Male	126 (32)		
Female	273 (68)		
Practice type^a^			
Solo practice	215 (54)		
Private clinic	64 (16)		
Public health center	86 (22)		
Private hospital	20 (5)		
Public hospital (non academic)	8 (2)		
Academic hospital	6 (1)		
Practice location^c^			
Jakarta	119 (30)		
Outside Jakarta but within Java island	167 (42)		
Outside java	113 (28)		
Participation in DM training^b^			
Yes	234 (64)		
No	165 (36)		
Number of DM patients seen in a week^c^		13.0 (15.9)	1-120
Proportion of DM patients among all patients seen^a^			
<10%	261 (66)		
10-30%	117 (29)		
>30%	21 (5)		
Awareness to DM consensus^a^			
Never knew	43 (11)		
Heard but never had a copy	138 (36)		
Had but never read the consensus	78 (20)		
Has read and implemented it	124 (33)		

Missing data: ^a:^<5%; ^b:^5-10%; ^c:^>10-15%.

**Figure 1:**
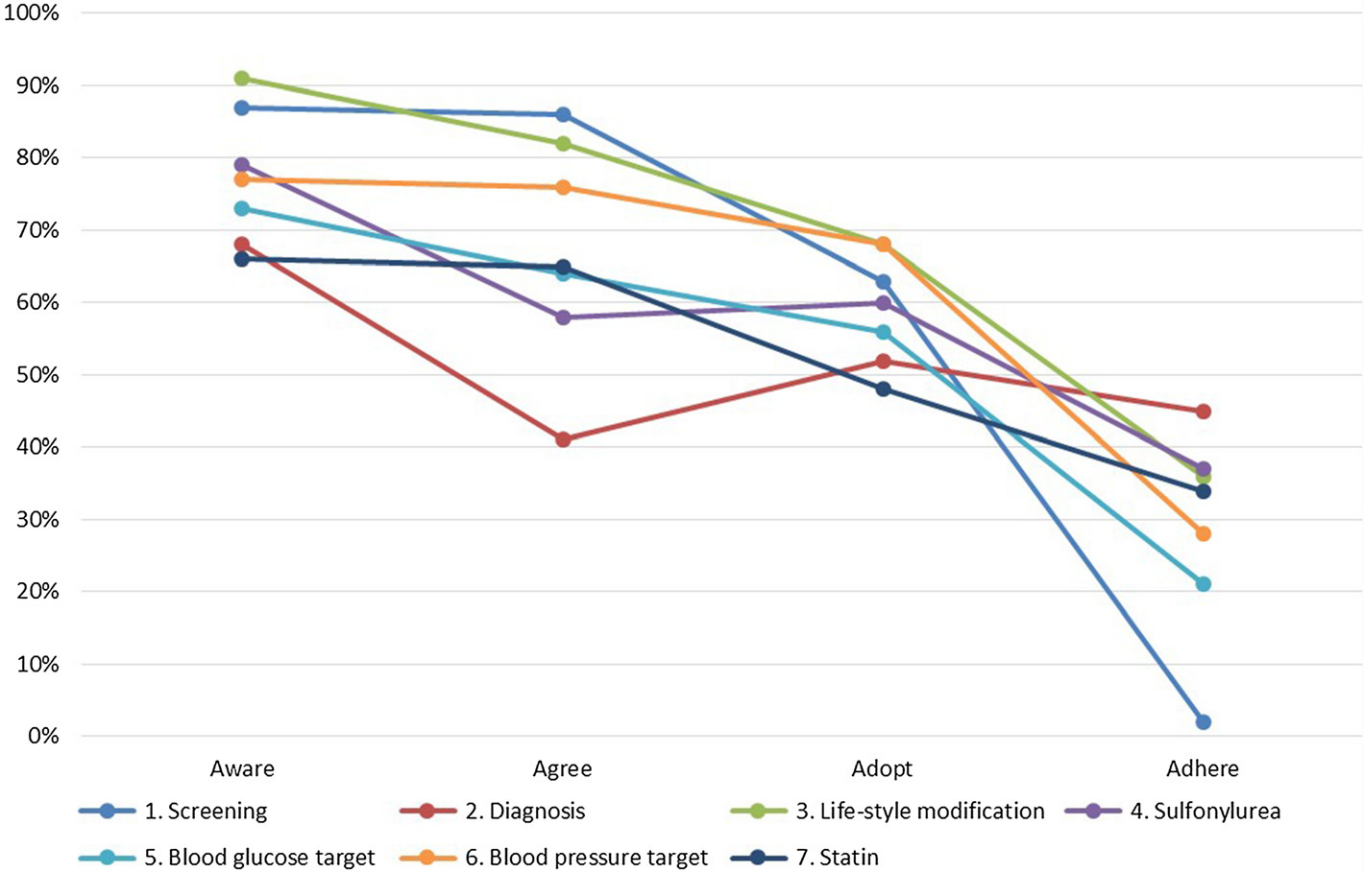
**Proportions of awareness, agreement, adoption, and adherence of GPs (n = 399) to selected recommendations from the Indonesian type 2 Diabetes Mellitus (T2DM) guidelines.** Proportions (%) were computed based on the total GPs responders. Missing data was: <5% for screening (all), diagnosis (all), lifestyle modifications (awareness, agreement and adoption), and adherence on sulfonylurea. 5-10% for adherence on lifestyle modification, sulfonylurea (awareness, agreement and adoption), blood glucose target (all), blood pressure target (all) and statin prescription (all).

Figure 1 shows the proportions of the responders who were aware of, agreed with, adopted and adhered to the recommendations of the Indonesian type 2 diabetes mellitus guidelines. Awareness of each recommendation varied between 66 to 91% while agreement with varied between 41 to 87%. The least aware (265/399, 66%) and least agreed (163/399, 41%) recommendation was on the use of random blood (plasma) glucose test to diagnose patients with classic diabetes symptoms.

Adoption varied between 48 to 68%. The least adopted was statin use in type 2 diabetes who are over 40 years old or have CVD risk (192/399, 48%). Adherence varied between 2 to 45%, while the least adherence was on the recommendation to perform type 2 diabetes screening in patients with any risk factor listed in the guideline (7/399, 2%).

The summary of the univariate associations (p ≤ 0.2) for six participant characteristics on adherence to the six recommendations can be found in Table 3 and the multivariate associations in Table 4. We were not able to investigate the presence of participant characteristics on the adherence to screening as the number of events was too small (7 events).

**Table 3:** Univariate associations (odds ratio and their 95% CI) between GPs (n = 399) characteristics and adherence to Indonesian type 2 Diabetes Mellitus (T2DM) guideline recommendations

	**Diagnosis**	**Lifestyle modification**	**Sulfonylurea for treatment**	**Blood glucose target**	**Blood pressure target**	**Statin prescription**
**Adherent participants: n (%)**	67 (45)	145 (36)	111 (37)	61 (21)	113 (28)	118 (34)
**Characteristics**						
**Years of practice**						
16 – 45*						
0 – 15	1.2 (0.8-1.7)^b^	1.4 (0.9-2.2)^c^	0.8 (0.6-1.3)^b^	0.8 (0.5-1.3)^c^	1.0 (0.7-1.6)^c^	0.7 (0.5-1.1)^c^
**Gender**						
Female*						
Male	1.1 (0.7-1.7)^a^	0.7 (0.5-1.2)^b^	1.3 (0.8-2.0)^a^	0.8 (0.5-1.4)^b^	0.8 (0.5-1.3)^b^	1.0 (0.6-1.6)^b^
**Practice type**						
Non solo practice*						
Solo practice	0.8 (0.6-1.2)^a^	0.7 (0.5-1.1)^c^	0.9 (0.6-1.3)^b^	1.0 (0.6-1.7)^c^	1.2 (0.8-1.9)^c^	1.0 (0.6-1.4)^b^
**Practice location**						
Outside Jakarta*						
Jakarta	0.9 (0.6-1.4)^c^	1.5 (1.0-2.3)^d^	1.0 (0.6-1.6)^c^	0.8 (0.5-1.4)^d^	1.1 (0.7-1.7)^d^	1.1 (0.7-1.7)^d^
**DM training**						
No*						
Yes	1.1 (0.7-1.7)^b^	1.3 (0.8-2.0)^d^	1.2 (0.8-1.9)^c^	1.0 (0.6-1.7)^d^	1.0 (0.6-1.6)^d^	1.1 (0.7-1.7)^d^
**Proportion DM patients**						
10% and above*						
<10%	1.0 (0.7-1.5)^b^	0.8 (0.5-1.2)^c^	0.9 (0.6-1.4)^b^	1.5 (0.9-2.5)^c^	1.1 (0.7-1.8)^c^	0.7 (0.5-1.1)^c^

Missing data: ^a:^0- < 5%, ^b:^5-10%, ^c:^>10-15%, ^d:^>15-19%.

*reference.

Adherence to screening was not included in the univariate analysis since the number of events were too small (seven events).

Associations which have p value <0.2,

• Life-style modification: years of practice, practice type, practice location.

• Blood glucose target: proportion of diabetes patients.

• Statin: years of practice, proportion of diabetes patients.

• Diagnosis, sulfonylurea for treatment and blood pressure target: none.

**Table 4:** Independent associations (multivariate odds ratio and their 95% CI) between GPs (n = 399) characteristics and adherence to Indonesian type 2 Diabetes Guidelines recommendations

	***Lifestyle modification***		***Blood glucose target***		***Statin prescription***	
***Characteristics***	**OR (95% CI)**	**p**	**OR (95% CI)**	**p**	**OR (95% CI)**	**p**
**Years of practice**						
16 – 45*						
0 – 15	1.2 (0.7-2.2)^c^	0.50	-		0.7 (0.4-1.1)^c^	0.07
**Gender**						
Female*						
Male	0.9 (0.5-1.4)^b^	0.51	-		-	
**Practice type**						
Non solo practice*						
Solo practice	0.9 (0.5-1.3)^c^	0.51	-		-	
**Practice location**						
Outside Jakarta*						
Jakarta	1.3 (0.8-2.1)^d^	0.29	-		-	
**DM training**						
No*						
Yes	-		-		-	
**Proportion DM patients**						
10% and above*						
<10%	0.8 (0.5-1.3)^c^	0.37	1.5 (0.9-2.5)^c^	0.16	0.7 (0.4-1.1)^c^	0.08

Missing data: ^a:^0- < 5%, ^b:^5-10%, ^c:^>10-15%, ^d:^>15-19%.

*reference.

Adherence to screening was not included in the multivariate analysis as the number of events were too small.

Adherence to recommendations on diagnosis, treatment and blood pressure target have no characteristic factors univariately associated (p-value ≤ 0.20) with them (see Table 3).

None of the participant characteristics were neither univariately nor multivariately associated with the recommendation on the diagnosis of diabetes (#2), Sulfonylurea for treatment (#4) and using blood pressure as treatment target (#6). During univariate regression analysis, years of practice, practice type and practice location, were associated with the recommendation on lifestyle modification (#3). However, none of these were retained during subsequent multivariate analysis. Only the proportion of diabetes patients was univariately associated with the recommendation on blood glucose as treatment target (#5).

During both univariate and subsequent multivariate analysis, adherence to the recommendation on statin prescription (#7) was found poor for responders with a practice prevalence of less than 10% diabetes patients (OR = 0.7, 95% CI 0.4;1.0, p = 0.08) and those practicing 15 years or less (OR =0.7, 95% CI: 0.4;1.0, p = 0.07).

## Discussion

This study shows that awareness and agreement of the GPs of the seven recommendations of the Indonesian type 2 diabetes mellitus guideline was quite high (66 to 91%). The high awareness of GPs and their familiarity with the guideline is most likely due to the extensive promotion and marketing efforts on the introduction of the Indonesian Society of Endocrinology guidelines. Despite this high awareness, a large number of GPs neither adopted nor adhered to the guideline recommendations. A practice prevalence of less than 10% diabetes patients and practicing 15 years or less were independently related with poor adherence to statin prescription.

### Awareness – agreement – adoption and adherence to the recommendations

There was high awareness among responders on the need for screening for type 2 diabetes risk factors among those without diabetes symptoms. Still, most of our responders waited until several cardiovascular risk factors emerged in the patient before they advised a screening test to the patient or they did not have a system to identify patients with risk factors. This was indicated by an extremely low (2%) adherence to the screening recommendation for identification of type 2 diabetes. This result is in contrast with findings among GPs in Switzerland about their adherence to the screening of diabetes which reach 83% [21].

We found the least agreement (41%) with the recommendation to use random blood (plasma) glucose in the diagnosis of diabetes in patients with classic diabetes symptoms. The majority of our responders believed it is more appropriate to examine patients with classic diabetes symptoms with a fasting plasma glucose test and 2-hour post-prandial plasma glucose test. Due to the fasting needed for these blood sugar tests, patients may delay or avoid the examination.

On statin prescription, we found that the level of awareness and adoption were the lowest (72% and 52%). With 32% adherence among our responders to the recommendation to prescribe statins, it is slightly higher than in a Seoul tertiary hospital (29%) [14] but much lower than the 68% adherence among Australian GPs [11]. Factors related to the adherence to the recommendation to prescribe statins in our study were more years of practice experience and larger proportion of diabetes patients the practice.

Adoption to guideline recommendations has been shown to be facilitated by the acquisition of the necessary knowledge and skills. High health costs for patients and practice, patient’s knowledge, expectations, compliance, motivation and support for recommendation, lack of materials, logistic support and time of health professionals, and high proportions of patients without insurance have been reported as barriers to guidelines adherence [16,17]. Adherence to actions recommended in guidelines may require practice organization. Besides, adherence is facilitated when tools are in place to put the recommendations into practice [22]. A systematic review [17] reported a median adherence of 36% (Inter Quartile Range 30 to 56%) to recommendations from various guidelines. Our data reveal that adherence to all the recommendations was quite low since less than 50% responders did not implement a system to promote and monitor recommendations in their practice.

Our findings on the low adherence of the recommendation on screening and diagnosis may explain why two thirds of the diabetes population in Indonesian remain un-diagnosed [2]. The generally low adherence to the recommendations of the Indonesian guideline may partly explain the finding by Soewondo et al. that 68% of patients in Indonesia diagnosed with diabetes were in poor glycemic control [6].

### Pattern of leakage

Based on a systematic review on the utilization of clinical guidelines [17] we expected to find a consistent pattern of ‘leakage’ , i.e. lower number of positive responders, over the four subsequent steps of the Pathman’s awareness-to-adherence model. In our study, adoption and adherence rates are generally progressively lower, except for the recommendations on diagnosis of diabetes and sulfonylurea treatment.

Our findings on the non-progressiveness of the recommendation on diagnosis of diabetes and sulfonylurea treatment deserve further consideration. As was stated previously, a majority of our responders believed that fasting and 2-hour post-prandial blood (plasma) glucose test are more appropriate than random blood (plasma) glucose in the diagnosis of diabetes in patients with classic diabetes symptoms. However, random blood glucose is more practical to be implemented in the practice so it is more adopted.

The Indonesian guideline recommends sulfonylurea as the drug of choice to manage hyperglycemia in normal and underweight type 2 diabetes patients. In all, 58% of our responders agree with this recommendation. In contrast to the Indonesian guidelines, the consensus statement of the American Diabetes Association and the European Association for the Study of Diabetes recommends simultaneous initiation of metformin and lifestyle intervention at diagnosis [10]. Familiarity with this ADA/EASD recommendation may cause some uncertainty, but metformin and other classes of blood glucose–lowering medications are not generally available in primary health centers [23]. This may explain why agreement with sulfonylurea as recommended treatment is lower than its adoption.

### Strengths and limitations of the study

Our study supports the usefulness of the awareness-to-adherence model and provides valuable information on the utilization of an important guideline in Indonesia. Still, some aspects of our study, notably the sampling method, response rate of the survey, missing data and the use of self-reporting questionnaire, need further consideration.

Obtaining representative samples from the large number of Indonesian GPs, exceeding 70,000, who are distributed over the archipelago possess challenges for our type of research.

Recruitment of responders among those attending a conference in Jakarta (capital city of Indonesia) was seen as more practical although it might not represent physicians who do not have opportunity to attend such meeting. However, our responders represent GPs from all parts of Indonesia. Our response rate is within the range of that of similar studies as reported by the systematic review of Mickan et al. [17].

Self-reporting is the most simple and inexpensive method of measuring adherence. However, it has several limitations including over-estimation due to recall bias and social desirability bias [24]. These drawbacks have been addressed through determination of specific time period in the questionnaire, assessment of specific behavior related to the recommendations, non-judgmental statements and confidentiality.

The awareness-to adherence model may help to identify GPs’ specific concerns with recommended practice changes. If uptake of a specific recommendation is low, qualitative approach to the concerns and barriers might be useful. Implementation of a quality assurance system which could further illustrate the care being received by diabetes patients in relation to the clinical outcomes is believed to be beneficial to promote adherence to guideline recommendations and increase the quality of diabetes care [25].

## Conclusions

Our study shows that high awareness of the guideline does not always lead to adoption nor adherence to its recommendations. The production and dissemination of guidelines alone is not sufficient to ensure that research evidence gets into practice. Improvement of clinicians’ awareness of, agreement with, and adoption to guidelines need to be incorporated in to strategies to improve guideline adherence.

## Competing interests

IS coordinated several diabetes projects of the Indonesian Society of Endocrinology. PS is the past president of the Indonesian Society of Endocrinology. YvdG, PG and GvdH have no competing interest.

## Authors’ contributions

GvdH (guarantor) and YvdG had the original idea, and with ISW and PG, designed the study. ISW and PS were responsible for data collection. ISW wrote the original draft of the manuscript, and all authors contributed to the concept and also all revised drafts of the manuscript. All authors read and approved the final manuscript.

## Pre-publication history

The pre-publication history for this paper can be accessed here:

http://www.biomedcentral.com/1471-2296/15/72/prepub
